# Rituximab treatment in adults with refractory minimal change disease or focal segmental glomerulosclerosis

**DOI:** 10.18632/oncotarget.21833

**Published:** 2017-10-15

**Authors:** Hong Ren, Li Lin, Pingyan Shen, Xiao Li, Jingyuan Xie, Xiaoxia Pan, Wen Zhang, Nan Chen

**Affiliations:** ^1^ Department of Nephrology, Institute of Nephrology, Ruijin Hospital, School of Medicine, Shanghai Jiaotong University, Shanghai, China

**Keywords:** rituximab, steroid-dependent, refractory FSGS, refractory MCD, clinical study, Immunology and Microbiology Section, Immune response, Immunity

## Abstract

Rituximab (RTX) may benefit patients with glomerular disease who suffer from focal segmental glomerular sclerosis (FSGS) or minimal change disease (MCD). Here, we have described our experience treating 6 FSGS and 9 MCD patients with steroid-dependent/refractory nephrotic syndrome (NS) with RTX. Patients received RTX (375 mg/m2) intravenously on days 1, 8, 23, and 29. During a median follow-up of 8 months (range, 3-36 months) after RTX administration, all patients achieved complete or partial remission. Relapses decreased by approximately 30-fold compared with the year preceding RTX treatment, and an 89.27% reduction in proteinuria was observed. Furthermore, RTX treatment could decrease medical costs by 76.52% compared with the costs associated with the long-term use (for 12-13 months) of steroids and immunosuppressive drugs.

In conclusion, RTX treatment was safe and effective for patients with refractory FSGS or MCD.

## INTRODUCTION

Minimal change disease (MCD) and focal segmental glomerular sclerosis (FSGS) are common causes of nephrotic syndrome (NS)[1, 2]. MCD accounts for 46.9% of the pediatric and 25.3% of the adult cases of primary NS in China [1, 2], In the USA, approximately 50–60% of adults with FSGS have NS [3, 4]. In MCD, initial therapy with oral steroids leads to a response rate of approximately 75%, but a high proportion of steroid-responsive patients experiences one or more relapses of NS [2]. In FSGS, up to 63% of patients treated with steroids achieve remission. Among those achieving complete or partial remission approximately 50% experience one or more relapses [5, 6]. Comorbidities secondary to steroid treatment may result in therapeutic dilemmas. Long-term complications of steroid therapy, such as reduced bone mineral density, dyslipidemia, embolism, impaired glucose tolerance, hypertension, and an increased risk of cardiovascular events, are commonly observed [7]. The aim of minimization of the cumulative steroid dosage, however, necessitates the use of alternative immunosuppressive agents. Despite the growing number of immunosuppressive agents that have proved to be effective, some severe cases remain difficult to treat and can lead to persistent relapsing NS. Immunosuppressive drugs including tacrolimus (TAC), cyclosporine (CsA), mycophenloatemofetil (MMF), and cyclophosphamide (CTX), are commonly used to lower or withdraw steroids in these patients.

The possibility of an effective and safe approach for patients with steroid-resistant NS (SRNS) or frequently relapsing NS (FRNS) emerged in 2004, when the B-cell-depleting rituximab (RTX) was reported to have induced the remission of proteinuria in a child with FRNS secondary to MCD [8, 9]. Moreover, RTX is a valuable option in the treatment of several connective tissue diseases and glomerular diseases such as idiopathic membranous nephropathy (IMN) [10-12]. In a small number of cases, adult patients with MCD and FSGS achieved remission after RTX treatment, suggesting that B-cell immunity could play a key role in the pathophysiologies of these diseases [13, 14]. Several mechanisms of RTX action have been suggested, including antibody-dependent/cell-mediated cytotoxicity, elimination by phagocytosis or cytokine-mediated cytotoxicity [15]. Nevertheless, in adult patients with MCD/FSGS, RTX was well tolerated and no life-threatening complications have been reported to date [8, 13]. However, no study has examined RTX treatment of adult Chinese patients with MCD or FSGS. Given that China is a developing country, the use of RTX to treat kidney diseases has not been incorporated into health care in China; therefore, this study investigated the therapeutic effects of RTX in patients with refractory MCD or FSGS.

## RESULTS

### Patient characteristics

At study entry, 15 patients who were 16-54 years of age (median, 25 years) were enrolled, with a female-to-male ratio of 0.36. In total, 9 patients had biopsy-proven MCD, and 6 patients had FSGS. All patients were severely nephrotic, with a time of relapse of 4±1.6, and the average disease duration was 76months(range, 12-156 months) (Table 1). All patients had failed prior immunosuppressive treatment with a combination of steroid and CTX (n=9), a combination of steroid and TAC (n=11), a combination of steroid and CsA (n=9), a combination of steroid and MMF (n=4), a combination of steroid and azathioprine (n=1), and/or multi-targeted therapy (n=7) (Table 1). In this study, 15 patients received RTX therapy, including 8 patients who received RTX in combination with low-dose steroids and TAC, 2 patients who received RTX in combination with steroids and CsA, 3 patients who received RTX in combination with steroids and MMF, 1 patient who received RTX in combination with steroids and azathioprine, and 1 patient who received RTX in combination with steroids.

**Table 1:** Main clinical and laboratory characteristics at baseline

Characteristic	
Number (n)	15
Age (yr)	25(16-54)
Female/male	0.36
Disease duration (months)	76(12-156)
Relapse number (n)	4±1.6
Oral steroids (n)	13
Cyclosporine (n)	9
Tacrolimus (n)	11
CTX (n)	9
MMF (n)	4
Azathioprine (n)	1
MCD (n)	9
FSGS (n)	6
Proteinuria, g/24 h	1.567(0.048-5.758)
Serum albumin, g/l	37(14-45)
Creatinine,µmol/l	63(42-78)
GFR(EPI, ml/min/1.73 m^2^)	128(93-135)

### Clinical outcomes

During the study period, 15 patients were followed for a median duration of 8 months (range, 3-36 months). The time course of urinary protein excretion, which was 1.567g/24h (range, 0.048-5.758g/24h)at baseline. By the end of 3 months, CR was achieved in 13 patients, and PR was achieved in 2 patients. The mean decrease in proteinuria from baseline to 3 months was 0.223g/24h, a reduction of 85.76%. The decrease in proteinuria was gradual and stable. The mean drop in proteinuria from baseline to 6 months was 0.158g/24h, a reduction of 89.91%. Among the 5 patients who completed 12 months of follow-up, 2 patients relapsed; these relapses occurred at 8 and 10 months of follow-up, with 24-hour proteinuria levels of 7.09g/24h and 14.369g/24h, respectively. Two patients achieved CR after 2 intravenous treatments with RTX (375 mg/m^2^), and their mean decrease in proteinuria from baseline to 12 months was 0.186g/24h, a reduction of 89.27%. Serum albumin was significantly increased among patients who achieved complete or partial remission. The remission rate of these patients at follow-up was 75-100% (Table 2). Based on a comparison of the year before RTX treatment and the first year of follow-up, during follow-up, total relapses decreased from 4 to 0.13, and the median number of relapses decreased to 0. Relapses decreased by 30-fold during the year of follow-up compared with the year preceding RTX treatment. No serious treatment-related adverse events were observed during follow-up.

**Table 2:** Clinical outcomes before and after RTX treatment

Time	Baseline	Month1	Month3	Month6	Month9	Month12
CR(%)	46.67	66.67	93.33	93.33	77.5	100
PR(%)	46.67	26.67	6.67	6.67	0	0
NR(%)	6.67	6.67	0.00	0	12.5	0
Adverse event(n)	0	0	0	0	0	0

### Steroid dosages before and after treatment with RTX

After RTX treatment, 5 patients stopped using steroids and immunosuppressive drugs for 6 months. Thus, 5 patients reached the main endpoint, and 10 patients reached the second endpoint. The median per-patient maintenance steroid dose decreased from 25.73 mg/day to 5.56 mg/day. Furthermore, the mean estimated GFR decreased from 115 to 113 ml/min /1.73 m^2^ (P=0.01). Treatment was well tolerated.

### CD19^+^ changes after RTX treatment

An initial depletion of CD19^+^ B-cells was observed in all patients (a median of 196 cells/ml at baseline versus 3 cells/ml at day 28). By 6 months, most patients continued to have CD19^+^ B-cell counts<5 cells/ml. However, no associations were observed between baseline proteinuria and changes in CD19^+^ B-cell depletion at 6 and 12 months. There were no significant changes in hemoglobin levels, total white blood cells, or platelet counts (Table 3). We found no correlations involving B-cell counts, their subsets, or changes in T-cell parameters.

**Table 3:** Main hematology parameters in 6 patients from rituximab administration (baseline) to the end of the study

Parameter	Baseline	3 months	6 months
Hemoglobin(g/l)	145(80-170)	141(80-157)	148(84-170)
White blood cell count	11.4(7.4-18.9)	7.57(4.5-20)	6.9(4.3-13.7)
Lymphocytes	3.2(1.5-13.5)	2.19(1.06-4.1)	2.03(1.1-28.6)
IgA(mg/dl)	199(90-340)	166(149-183)	207(81-291)
IgG(mg/dl)	844(218-2320)	790(730-851)	1185(470-1650)
IgM(mg/dl)	129(22-254)	89(21-157)	112(28-246)
B cell subset analysis in peripheral blood mononuclear cells			
CD19^+^ (B cells)	196(16-595)	5.8(1-290)	4.1(1.77-18)
T cell subset analysis in peripheral blood mononuclear cells			
CD3^+^T cells (%)	9.85(1.3-36.3)	82.75(73.3-93.8)	84.05(77.7-92.7)
CD3^+^CD4^+^ (helper) T cells(%)	78.90(7.1-83.8)	39(18.6-42.2)	46.45(43.6-48.2)
CD3^+^CD8^+^ (suppressor) T cells(%)	37.65(5.9-55.2)	42.25(35.7-54.6)	34.65(31.1-38.2)

### Health economy

In this study, the average medical cost for the 15 patients was $64,224 (range, $17,460-$142,857) prior to RTX treatment; the cost of RTX treatment was $15,079, which was much cheaper than the cost of the long-term use of steroids and immunosuppressive drugs, and represented a cost reduction of 76.52%.

## DISCUSSION

RTX has shown potential role in treatment of many autoimmune diseases, including systemic vasculitis and lupus erythematosis, as well sporadic cases of MCD and FSGS. This study represented the largest prospective analysis to date that evaluated RTX in the treatment of Chinese patients with FRNS with 8 months (range, 3-36 months) of follow-up. In this investigation, we found that RTX significantly reduced the frequency of relapses in 15 patients with frequently relapsing or steroid-dependent NS. Relapses decreased by approximately 30-fold during follow-up compared with the year preceding RTX treatment, and an 89.27% reduction in proteinuria was observed. The therapeutic effects of RTX in FSGS and MCD were similar. A number of reports have reported using RTX in children with NS [16-18]. RTX therapy could significantly achieve a higher rate of complete remission and reduce the occurrence of proteinuria [19]. A recent study showed that 54.5%(12/22) patients aged from 6.2-25 years responded to rituximab [20]. In adult FSGS and MCD, RTX reduced the number of relapses per year from 1.3 (0–9) relapses prior to treatment compared to 0 (0–2) after therapy [8]. The result of our study about the remission rate in frequent relapse FSGS and MCD was similar to other studies.

In general, the mode of RTX administration was not uniform. Treatment of RTX was quite variable in different studies: a single course of rituximab consisted of 1 to 4 consecutive pulses of 375 mg/m^2^ at weekly or 1000mg dose dose on days 1 and 15 [8, 15, 19]. After therapy, the number of relapses reduced from 1.3 (0–9)/year to 0 (0–2) /year (p < 0.001) and proteinuria decreased from 2.43 (0–15) g/day to 0 (0–4.89) g/day (p < 0.001) [8]. Ruggenenti et al argue for administering 375 mg/m^2^, as a single dose with circulating B cells are measured 24 hours and 1 week after Rituximab administration in order to drive RTX administration [14]. Kamel El-Reshaid et al reported that treatment with 4-weekly infusions of 500mg resulted in amelioration of NS in 17 of the 18 patients with FSGS, but proteinuria reappeared by 8 to 12 months [18, 21]. The protocol of RTX was that patients received RTX (375 mg/m^2^) intravenously on days 1, 8, 23 and 29. The reason as follows: 1) We determined that the terminal half-lives of RTX were 11.5 days in patients with MN and 18.0 days in patients with RA [10]. Given that almost all of these patients exhibited steroid-dependent FSGS/ MCD with moderate to nephrotic-range proteinuria, we believed the pharmacokinetic effects of RTX in these patients would persist for 11.5-18 days. 2) According to a previous report, RTX will be lost via urinary excretion [10], half-life period would shorter than 11.5 days. So we regarded 4-weekly consecutive pulses of 375 mg/m2 RTX ensured the drug concentration reached an appropriate peak. 3) The reasons why we prolonged interval to 2 weeks between the second RTX doses and third as followed: After 2 times treatment, patients’ proteinuria decreased significantly. So less RTX leakage from the kidney and longer pharmacokinetic occur, and the interval after the second treatment can be extended to 11.5-18 days.

The results of our study strongly support a role for RTX in the treatment of FSGS/MCD and offer a more selective therapeutic approach. We observed CR or PR in 75-100% of patients at 1 year. Consistent with previous reports, RTX was well tolerated, and infusion reactions were the most common side effects. Furthermore, this study determined that the cost of RTX treatment was no greater than that associated with the long-term use of traditional treatments for FSGS and MCD. In fact, RTX treatment could reduce spending for frequently relapsing patients.

The limitations of this study include the lack of a randomized design, the absence of a placebo control, and a limited sample size. Furthermore, follow-up beyond 3 years is required to assess the durability and safety of responses to RTX.

In conclusion, RTX appears to effectively decrease relapses, and RTX treatment can achieve CR or PR of proteinuria in patients with FSGS or MCD in a cost-effective manner. Our results are particularly remarkable given that all patients had failed their therapies prior to treatment with RTX. We found that intravenous RTX (375 mg/m^2^) on days 1, 8, 23, and 29 was an appropriate dosing approach for steroid-dependent/refractory FSGS and MCD. More prospective randomized controlled trials of RTX are needed to truly assess this drug’s efficacy and durability compared with traditional specific immunosuppressive treatments.

## MATERIALS AND METHODS

### Patient population

This study was a single-center prospective study. Before treatment, we obtained informed consent from the included patients and approval from the scientific and ethics committee of our hospital.

We recruited 15 patients with FRNS who had renal biopsy-proven FSGS or MCD, exclude inheritance and secondary (Supplementary Figures 1-3, Supplementary Table 1), exhibited evidence of long-term treatment with immunosuppressive drugs (CsA, TAC, CTX, MMF or azathioprine), and had a GFR (glomerular filtration rate) higher than 60 ml/min calculated using the CKD-EPI (chronic kidney disease epidemiology collaboration) equation. FRNS was defined as 2 or more relapses within 6 months of the initial response or 4 or more relapses in any 12-month period. The exclusion criteria were pregnancy, infections (including hepatitis C, hepatitis B and HIV), diabetes mellitus, malignancy, glomerulonephritis other than FSGS or MCD, and any systemic disease.

### Treatment protocol and follow-up

FRNS patients with biopsy-proven FSGS or MCD were enrolled. They were treated with steroids, RTX and low-dose immunosuppressive agents. RTX (375 mg/m^2^) was administered intravenously on days 1, 8, 23 and 29. To minimize infusion reactions, patients were orally premedicated with acetaminophen and diphenhydramine hydrochloride. In addition, methylprednisolone (40 mg, intravenous) was administered prior to the first RTX infusion. B-cell depletion was defined as a CD19^+^ count<5 cells/µl. One month after the final infusion of RTX, the doses of both steroids and immunosuppressive drugs were tapered each month until these drugs were successfully discontinued or evidence of a relapse of NS was observed.

Before treatment, a kidney biopsy was performed for all patients. Patients’ baseline clinical characteristics and laboratory data were reviewed after the screening visit. The following data were collected and evaluated at study entry and at months 1, 3, 6, 9, and 12: clinical features, blood pressure, side effects, findings from routine examinations of blood and urine, liver and renal function, plasma electrolyte content, 24-hour protein excretion, serum cholesterol, serum glucose, and serum immunoglobulins (IgG, IgM, IgA, IgE). The medical costs associated with RTX and with other therapies used prior to RTX were also assessed for each patient to analyze whether RTX therapy was economically advantageous.

### Outcome measures

The main outcome measures were the percentage of patients who achieved treatment withdrawal with no evidence of relapse and the percentage of patients who maintained complete or partial remission after treatment withdrawal. The secondary endpoints included monthly changes in protein and the number of patients with PR (partial remission) or CR (complete remission) at 6 and 12 months. CR was defined as proteinuria<0.3 g/24 h, PR was defined as proteinuria<3.5 g/24 h and a <50% reduction in peak proteinuria, and nonresponse was defined as a <50% reduction in peak proteinuria. Any patient who achieved CR or PR was regarded as a treatment success.

### Statistical analyses

Monthly changes were tested against zero using paired t-tests. Associations between urinary protein and immune response were assessed using Pearson’s correlation coefficients for two-sided hypothesis tests performed with a significance threshold of 0.05. Statistical analyses were conducted using SPSS, version 20.

## SUPPLEMENTARY MATERIALS FIGURES AND TABLE


