# Supplementary material for: Rituximab treatment in adults with refractory minimal change disease or focal segmental glomerulosclerosis
Source: Oncotarget. 2017 Oct 15;8(55):93438–43. doi: 10.18632/oncotarget.21833 (PMC5706808; doi:10.18632/oncotarget.21833)
Supplement: Supplementary file 1 [file oncotarget-08-93438-s001.pdf]

# Rituximab treatment in adults with refractory minimal change disease or focal segmental glomerulosclerosis

## SUPPLEMENTARY MATERIALS

A total of 26 exons (INF2 exon 2-4, TRPC6 exon 1-13 and ACTN4 exon 1-10) were screened for mutations in genes of INF2, TRPC6 and ACTN4 in 15 patients. RESULTS: (1) No new mutation sites were found (Fig. 1 - Fig. 3). (2) There were 9 deletions in the template, 7 in the intron region and 2 in the exon region, all of which were reported SNPs (Table 1).

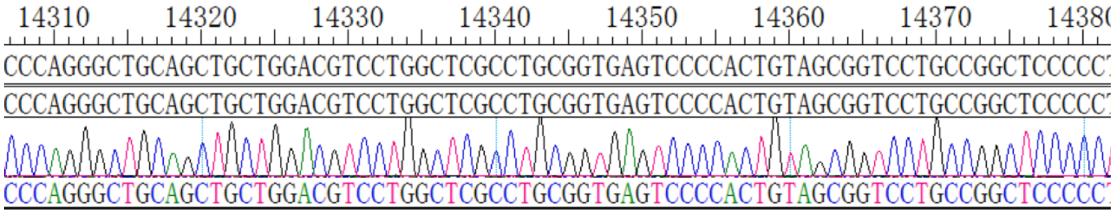


**Supplementary Figure 1: New mutation sites not detected in INF2**

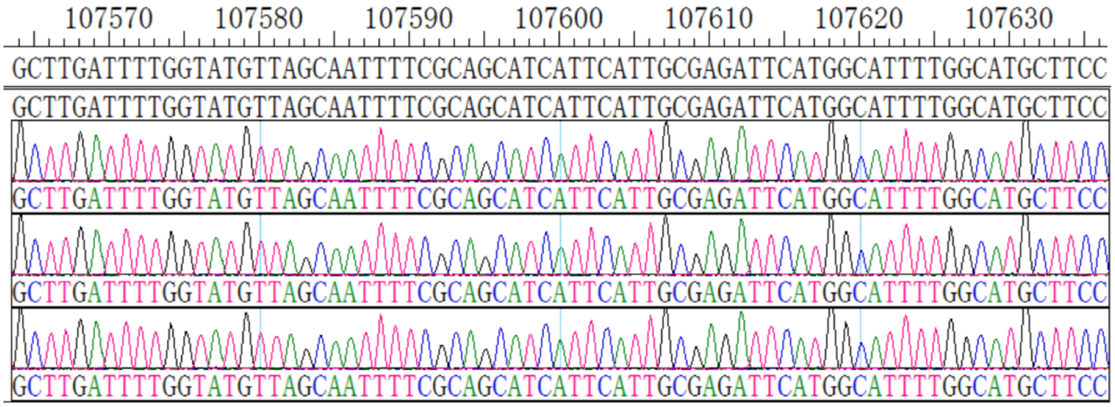


**Supplementary Figure 2: New mutation sites not detected in TRPC6**

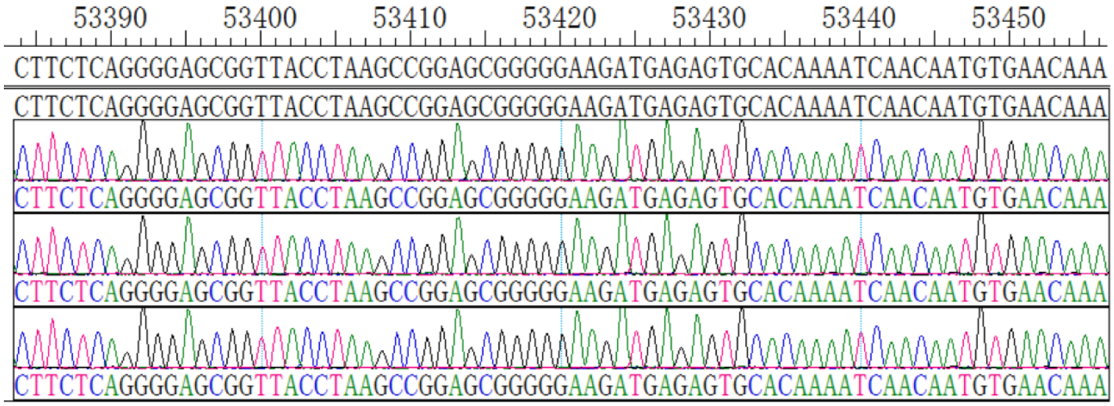


**Supplementary Figure 3: New mutation sites not detected in ACTN4**

**Supplementary Table 1: 9 SNPs were found**

| Gene | Exon | Mutation | SNP ID | Chromosome | Global MAF |
|---|---|---|---|---|---|
| ACTN4 | 1 | 162+61T>C | rs2303040 | 19:38647968 | C=0.2728/1366 |
| ACTN4 | 2 | 277+29G>C | rs2112649 | 19:38700743 | G=0.3293/1649 |
| ACTN4 | 5 | 554T>C | / | 19:38706113 | / |
| ACTN4 | 5 | 546C>T | rs3745859 | 19:38706105 | T=0.3393/1699 |
| ACTN4 | 5 | 572+11G>A | rs60710743 | 19:38706142 | A=0.0002/1 |
| ACTN4 | 8 | 734-54C>T | rs12984794 | 19:38710203 | T=0.0691/346 |
| INF2 | 4 | 668-90G>A | rs7145815 | 14:104703826 | A=0.1316/659 |
| INF2 | 4 | 668-78T>G | rs7146817 | 14:104703838 | G=0.1322/662 |
| INF2 | 4 | 668-31G>A | rs7145851 | 14:104703885 | A=0.1314/658 |
